# NGF/TRKA Decrease miR-145-5p Levels in Epithelial Ovarian Cancer Cells

**DOI:** 10.3390/ijms21207657

**Published:** 2020-10-16

**Authors:** Maritza P. Garrido, Ignacio Torres, Alba Avila, Jonás Chnaiderman, Manuel Valenzuela-Valderrama, José Aramburo, Lorena Oróstica, Eduardo Durán-Jara, Lorena Lobos-Gonzalez, Carmen Romero

**Affiliations:** 1 Laboratorio de Endocrinología y Biología de la Reproducción, Hospital Clínico Universidad de Chile, Santiago 8380456, Chile; mgarrido@hcuch.cl (M.P.G.); ignacio.torres.p@ug.uchile.cl (I.T.); jose.aramburo@ug.uchile.cl (J.A.); 2 Departamento de Obstetricia y Ginecología, Facultad de Medicina, Universidad de Chile, Santiago 8380453, Chile; 3 Centro de Medicina Regenerativa, Facultad de Medicina, Clínica Alemana-Universidad del Desarrollo, Santiago 7710162, Chile; albaavila@gmail.com (A.A.); eduranj@udd.cl (E.D.-J.); 4 Programa de Virología, Instituto de Ciencias Biomédicas, Facultad de Medicina, Universidad de Chile, Santiago 8380453, Chile; jchnaiderman@med.uchile.cl; 5 Laboratorio de Microbiología Celular. Instituto de Investigación e Innovación en Salud. Facultad de Ciencias de la Salud, Universidad Central de Chile, Santiago 8320000, Chile; manuel.valenzuela@ucentral.cl; 6 Centro de Investigación Biomédica (CIB), Facultad de Medicina, Universidad Diego Portales, Santiago 8370007, Chile; lorenaorostica@gmail.com

**Keywords:** microRNA-145, NGF, TRKA, epithelial ovarian cancer, c-MYC, VEGF

## Abstract

Nerve Growth Factor (NGF) and its high-affinity receptor tropomyosin receptor kinase A (TRKA) increase their expression during the progression of epithelial ovarian cancer (EOC), promoting cell proliferation and angiogenesis through several oncogenic proteins, such as c-MYC and vascular endothelial growth factor (VEGF). The expression of these proteins is controlled by microRNAs (miRs), such as miR-145, whose dysregulation has been related to cancer. The aims of this work were to evaluate in EOC cells whether NGF/TRKA decreases miR-145 levels, and the effect of miR-145 upregulation. The levels of miR-145-5p were assessed by qPCR in ovarian biopsies and ovarian cell lines (human ovarian surface epithelial cells (HOSE), A2780 and SKOV3) stimulated with NGF. Overexpression of miR-145 in ovarian cells was used to evaluate cell proliferation, migration, invasion, c-MYC and VEGF protein levels, as well as tumor formation and metastasis in vivo. In EOC samples, miR-145-5p levels were lower than in epithelial ovarian tumors. Overexpression of miR-145 decreased cell proliferation, migration and invasion of EOC cells, changes that were concomitant with the decrease in c-MYC and VEGF protein levels. We observed decreased tumor formation and suppressed metastasis behavior in mice injected with EOC cells that overexpressed miR-145. As expected, ovarian cell lines stimulated with NGF diminished miR-145-5p transcription and abundance. These results suggest that the tumoral effects of NGF/TRKA depend on the regulation of miR-145-5p levels in EOC cells, and that its upregulation could be used as a possible therapeutic strategy for EOC.

## 1. Introduction

Among the malignancies of the female reproductive system, epithelial ovarian cancer (EOC) is the leading cause of death [1,2,3]. Given that patients with EOC have non-specific symptoms [4], it is diagnosed in late stages, with poor survival rates [5]. In addition, current therapies are non-specific for cancer cells and have shown modest results and relapse rates of 70–80% within the first 2 years [6]. Therefore, a better understanding of the pathophysiology of this disease will allow the development of new therapeutic strategies.

A hallmark of cancer cells is sustained proliferative signaling [7], such as the over-expression of growth factors, which promote cell proliferation and angiogenesis in EOC [8,9,10,11], two pivotal processes in cancer development and progression. Our previous reports have shown that nerve growth factor (NGF) and its high-affinity receptor TRKA increase mRNA and protein levels during EOC progression, being higher in advanced stages of this disease [9]. NGF enhances proliferation of EOC cells due to increased levels of the c-MYC transcription factor [12], which regulates the expression of cyclins and many oncogenic proteins related to the cell cycle and cell death [13]. On the other hand, NGF is considered a direct and indirect angiogenic factor, because it acts directly on endothelial cells (which express the TRKA receptor), increasing their proliferation, migration and angiogenic potential [14]. In addition, one of the most studied angiogenic factors in ovarian cancer is vascular endothelial growth factor (VEGF), whose expression is increased by NGF/TRKA in EOC cells [9,15]. NGF/TRKA activates some signaling pathways (PI3K/AKT and MAPK/ERK) [16] associated with higher levels of oncoproteins, such as c-MYC and VEGF. Moreover, NGF/TRKA signaling pathways are associated with the modulation of micro-RNA (miR) transcription and its abundance in cancer cells [17,18,19].

Several studies have shown that ovarian cancer cells can upregulate most oncogenic proteins by altering miR levels [20,21]. miRs are evolutionarily conserved non-coding RNA of 18–25 nucleotides that drive gene silencing either by mRNA degradation or by preventing mRNA translation [22]. Interestingly, one miR can regulate the translation of hundreds of cell proteins [23,24]. Therefore, miRs constitute an important system of post-transcriptional regulation in eukaryotic cells. Because miRs are involved in the regulation of development, differentiation, apoptosis and cell proliferation [25,26,27], some of them are considered oncogenes (oncomiRs) or tumor suppressor miRs (oncosuppressors). In EOC, miRs such as Let-7, the miR-200 family, miR-17-92, miR-21, miR-145 and miR-23b have been found to be altered [28,29,30].

Some studies have shown that miR-145 functions as a tumor suppressor, whose decrease is related to poor prognosis in several cancers, such as prostate, breast, gastric and colorectal cancer [31,32,33,34]. In colon and breast cancer cells, miR-145 reduces the levels of several oncoproteins, such as c-MYC and VEGF [35,36], decreasing cell proliferation and the angiogenic potential of cancer cells. In EOC, miR-145 was proposed as a diagnosis marker and predictor of disease outcome [37,38]. In addition, recent evidence shows that downregulation of miR-145 levels in EOC samples induces an increase in several oncoproteins and thus, promotes EOC progression [39,40,41,42,43]. The main knowledge about miR-145 in ovarian cancer comes from miR-145-5p, so we decided to focus our study on miR-145-5p and its relationship with NGF/TRKA action in EOC cells.

Since NGF/TRKA has an important role in EOC and can regulate the expression of many oncogenic proteins, including c-MYC and VEGF, the purpose of this study was to evaluate whether NGF/TRKA modulates miR-145 levels in EOC cells. Additionally, we studied the anti-tumoral proprieties of miR-145 through its upregulation in both in vitro and in vivo models.

Our results show that miR-145 levels decrease during EOC progression and that miR-145 upregulation decreases cell proliferation and c-MYC and VEGF levels in ovarian cells, as well as tumor size and the malignant presence of ascites in a mouse model. An in silico analysis showed that miR-145 could regulate several proteins downstream of NGF/TRKA signaling. As expected, NGF stimulation decreased miR-145 transcription and miR-145 abundance in EOC cells, in a TRKA-dependent manner. These results suggest that, at least in part, proliferative and angiogenic mechanisms of NGF are dependent on miR-145 regulation in EOC cells and that miR-145 up-regulation could be considered as a possible therapeutic strategy in EOC.

## 2. Results

### 2.1. miR-145-5p Levels Decrease during EOC Progression

**Figure 1:**
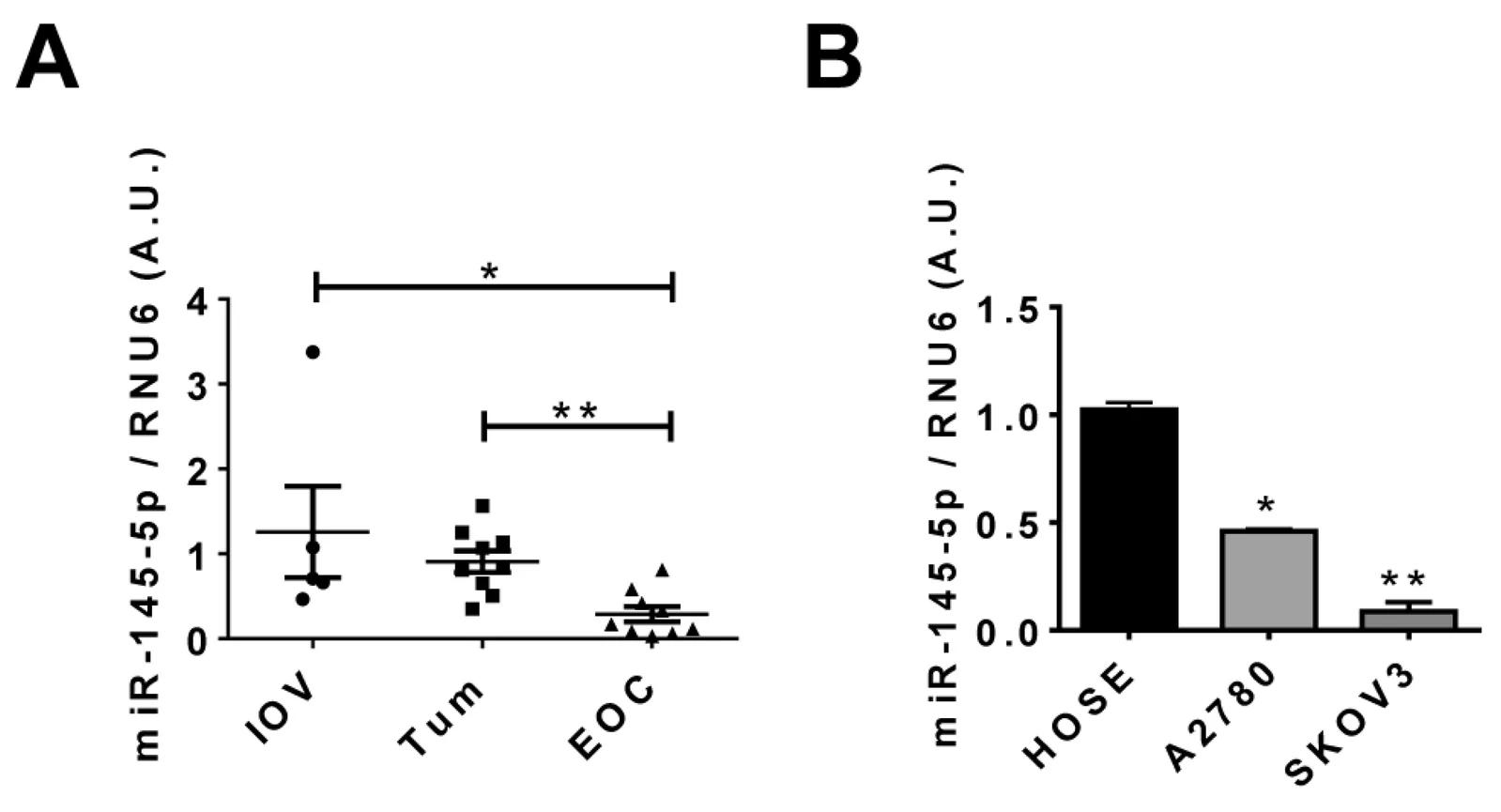
miR-145-5p levels in ovarian biopsies and ovarian cell lines. (**A**) miR-145-5p levels were measured by qRT-PCR in ovarian biopsies from: inactive ovaries (IOV, from post-menopausal women), epithelial ovarian tumors (Tum) and epithelial ovarian cancer (EOC). *N* = 5 (for IOV) and 9 (for Tum and EOC). * = *p* < 0.05 and ** = *p* < 0.01 as indicated, according to the Kruskal–Wallis test and Dunn’s post-test. (**B**) miR-145-5p levels (measured by qPCR) in HOSE, A2780 and SKOV3 cells, normalized to values obtained with HOSE cells. *N* = 4 (for HOSE and A2780 cells) and *N* = 8 for SKOV3 cells. U6 small nuclear RNA (RNU6) was used as housekeeping miR. * = *p* < 0.05 and ** = *p* < 0.01, respect to HOSE cells (Kruskal–Wallis test and Dunn’s post-test). A.U.: arbitrary units. Results are expressed as standard error of mean (SEM).

miR-145-5p levels were quantified in inactive ovaries (IOV, obtained from post-menopausal women), epithelial ovarian tumors (Tum) and serous epithelial ovarian cancer biopsies (EOC). The results show that miR-145-5p levels decreased during EOC progression, being lower in EOC biopsies compared with IOV or Tum (*p* < 0.05 and *p* < 0.01, respectively; Figure 1A). To validate the in vitro models, baseline levels of miR-145-5p were measured in the ovarian cell lines HOSE (immortalized epithelial surface ovarian cells, with growth and morphologic features that resemble the ovarian surface epithelium [44]), A2780 (EOC cells from primary origin [45]) and SKOV3 (EOC cells from ascites source [46]). As expected, miR-145 levels were lower in A2780 and SKOV3 cells, compared to HOSE cells (*p* < 0.05 and *p* < 0.01, respectively; Figure 1B).

### 2.2. Transient Over-Expression of miR-145 Decreases Cell Proliferation of Ovarian Cells

**Figure 2:**
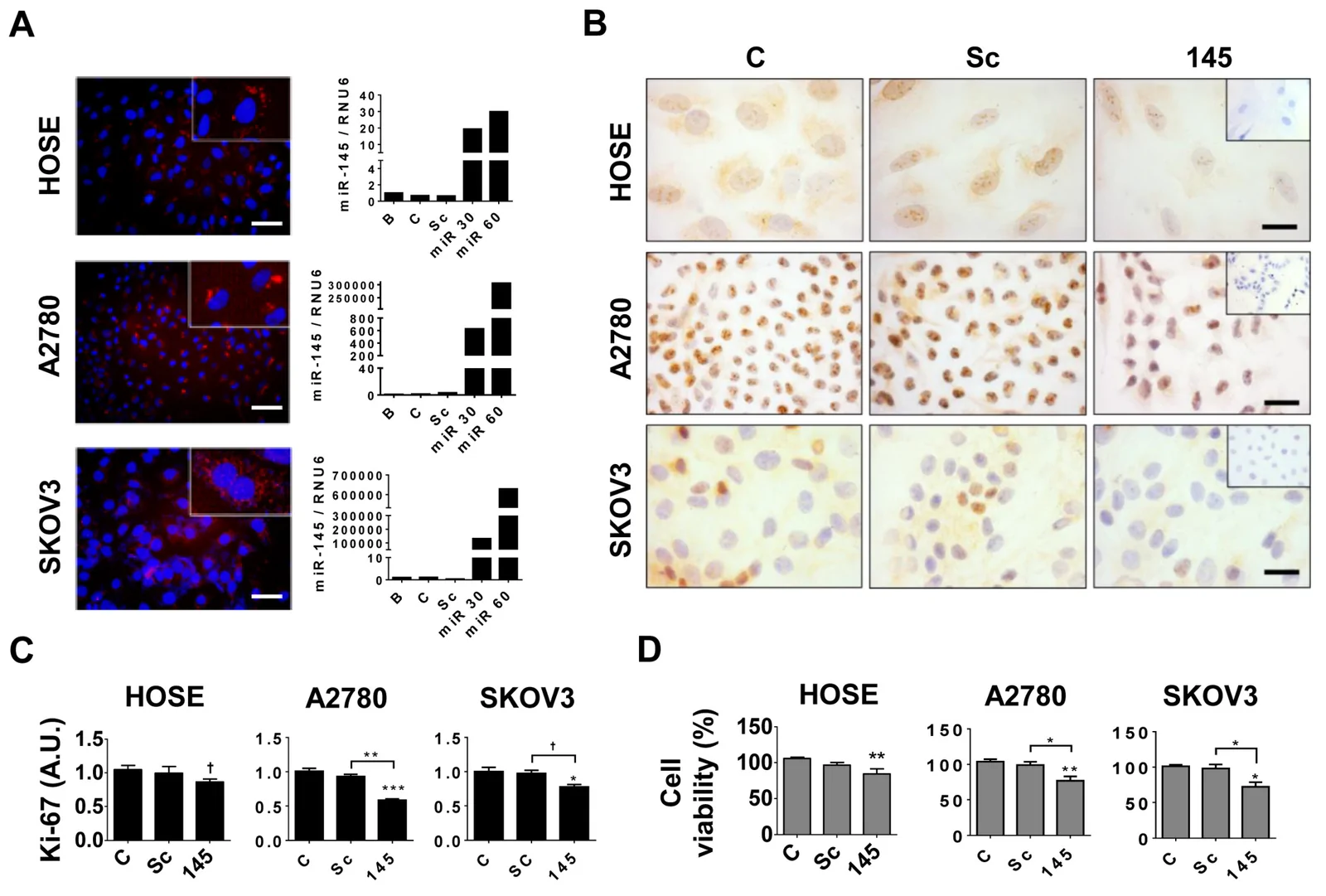
Effect of miR-145 upregulation in cell proliferation of ovarian cells. Ovarian cells were transfected with miR-145 (145), a scrambled sequence (Sc) or none (C, control) using Lipofectamine 2000 and cell proliferation was measured by the MTS assay and Ki-67 immunodetection. (**A**) miR-145 fluorescence (red) in ovarian cells after transfection and miR-145 levels in transfected ovarian cells. B: basal condition (without stimuli). miR 30 and miR 60: Cells transfected with miR-145 30 and 60 μM, for 48 h. (**B**) Representative pictures of Ki-67 immunodetection (brown) of ovarian cells under the respective conditions. Harris’ Hematoxylin (blue) was used as a counterstain. Right corner: negative control (without primary antibody). Bar = 50 μm. (**C**) Analysis of Ki-67 immunocytochemistry in ovarian cells. *N* = 4 (4–10 pictures per condition were analyzed). (**D**) Cell viability of ovarian cells (MTS assay) under the respective treatments. *N* = 4 (duplicate). For (**C**) and (**D**): * = *p* < 0.05, ** = *p* < 0.01 and *** = *p* < 0.001, compared with the control condition, or as indicated (Kruskal–Wallis test and Dunn’s post-test). † = *p* < 0.05, compared with the control condition, or as indicated, according to the Mann Whitney test. Results are expressed as standard error of mean (SEM).

Ovarian cells were transfected with synthetic miR-145, as described in the methodology section, and changes in cell proliferation were assessed by Ki-67 immunodetection using a 3-(4,5-dimethylthiazol-2-yl) (MTS) assay. The transfection efficiency of miRs was evaluated by qPCR and observed by fluorescence (red fluorescence of internal mark of miR-145), obtaining a significant increase in miR-145 from basal levels in all ovarian cell lines (Figure 2A). The results show that miR-145 over-expression decreased Ki-67 immunodetection in HOSE, A2780 and SKOV3 cells, showing a strong effect in both EOC cell lines (*p* < 0.05, *p* < 0.001 and *p* < 0.05, respectively; Figure 2B,C). In a similar manner, miR-145 over-expression decreased cell viability in the three ovarian cell lines (*p* < 0.01 for HOSE and A2780 cells and *p* < 0.05 for SKOV3 cells; Figure 2D).

### 2.3. Over-Expression of miR-145 Decreases Migration and Invasion of EOC Cells

**Figure 3:**
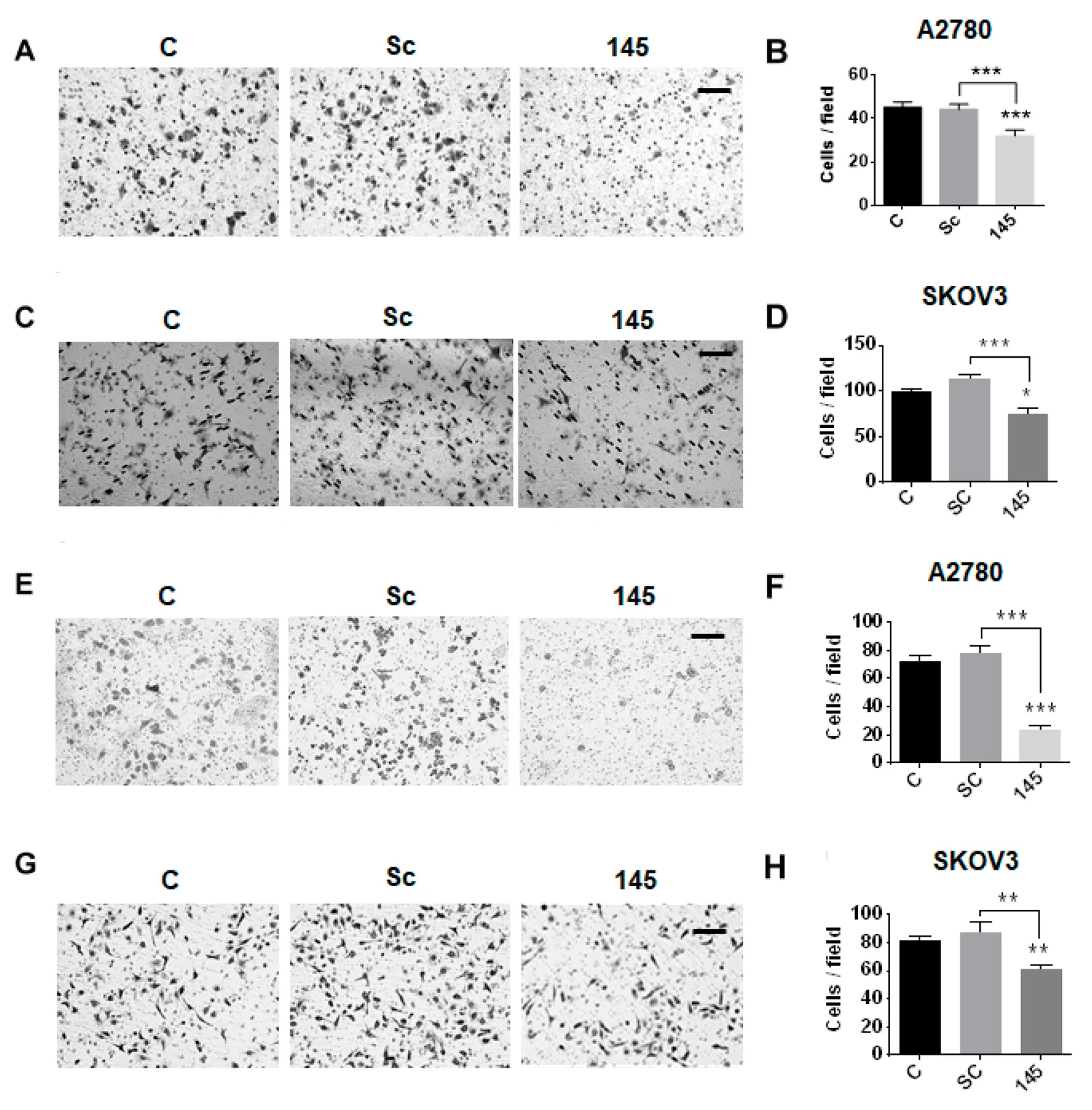
The over-expression of miR-145 decreases the migration and invasion ability of EOC cells. EOC cells were transfected with miR-145 (145), a scrambled sequence (Sc) or none (C, control), using Lipofectamine 2000 (levels of miR-145 after transfection are shown in Figure 2) and cell migration and invasion were assessed as described in the Methodology section. (**A**,**C**) Representative pictures of A2780 and SKOV3 cells after the migration assays (stain: crystal violet). (**B**,**D**) The quantification of the migration assays (cells that crossed the membrane/field) of the respective experimental groups. (**E**,**G**) Representative pictures of A2780 and SKOV3 cells after the invasion assays (stain: toluidine blue). Bar = 100 µm. (**F**,**H**) The quantification of the invasion assays (cells that crossed the membrane/field) of the respective experimental groups. *N* = 4 (3–5 pictures per condition were analyzed). Bar = 100 µm. * = *p* < 0.05, ** = *p* < 0.01 and *** = *p* < 0.001 (Kruskal–Wallis test and Dunn’s post-test), either with respect to the control condition, or as indicated. The results are expressed as standard error of mean (SEM).

Another key feature of EOC cells is their high migration and invasion potential, promoting EOC dissemination. To evaluate whether miR-145 modulates these processes in EOC cell lines, two functional assays were performed, using A2780 and SKOV3 EOC cells. The results show that over-expression of miR-145 significantly decreases cell migration of A2780 and SKOV3 cells, compared with the scrambled and control conditions (*p* < 0.001 and *p* < 0.05 respectively; Figure 3A–D), as well as their invasion ability, compared with scrambled and control (*p* < 0.001 and *p* < 0.05 respectively, in A2780 cells and *p* < 0.01 and *p* < 0.01, respectively in SKOV3; Figure 3E–H).

### 2.4. Over-Expression of miR-145 Decreases Presence of Ascites and Tumor Size of EOC Xenografts

**Figure 4:**
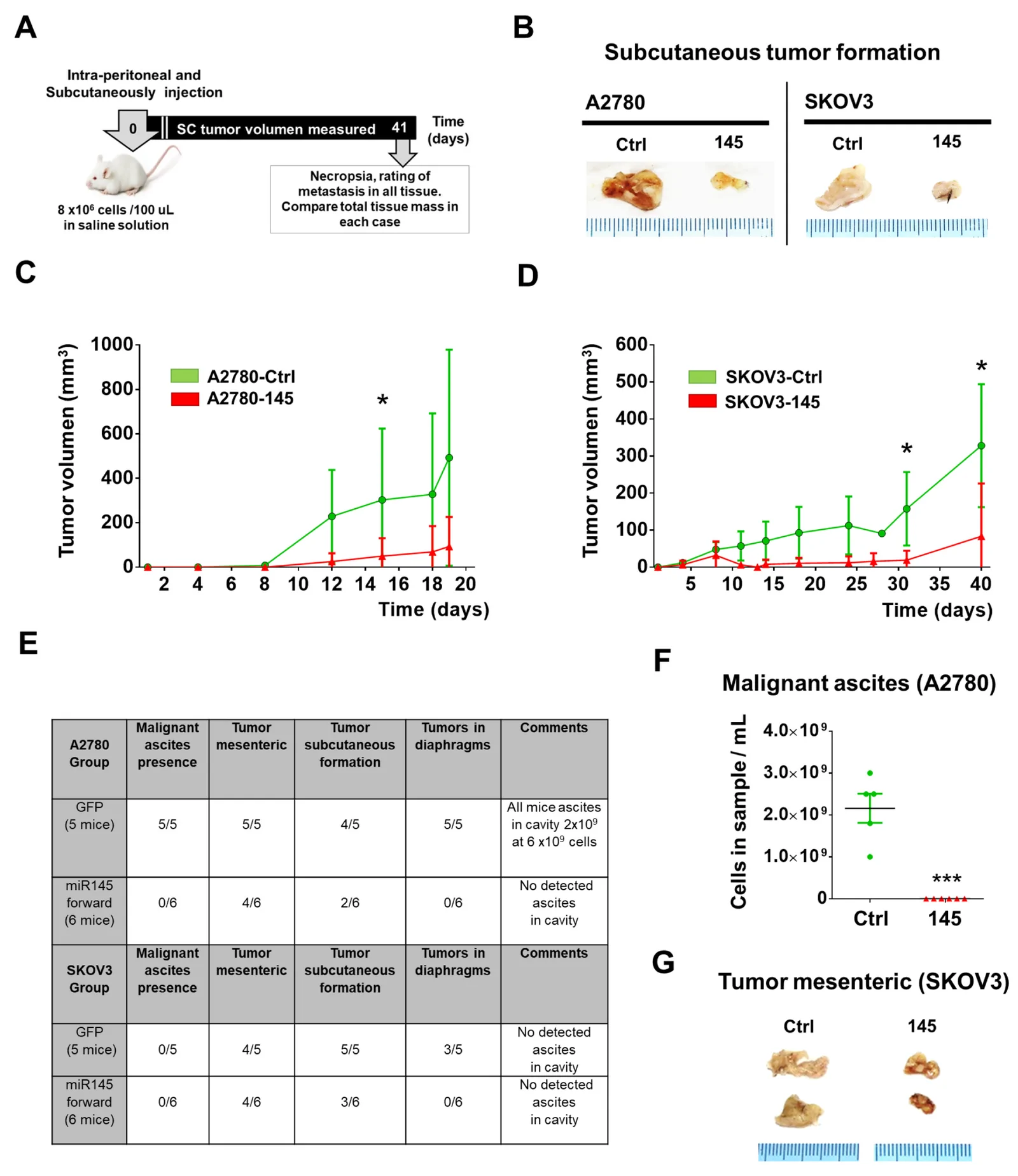
Over-expression of miR-145 decrease tumorigenic and metastatic behavior in an in vivo mouse model of EOC. EOC cells (A2780 and SKOV3) were transduced with lentiviral vectors as described in the methodology section, to obtain control animals with EOC (A2780-Ctrl and SKOV3-Ctrl) and animals with tumors that over-expressed miR-145 (A2780-145 and SKOV3-145). (**A**) Scheme of the carcinomatosis metastatic model using NOD/SCID mice, as described in the methodology section. (**B**) Photographs of representative experiments showing subcutaneous tumor growth in the lateral flank of mice (in order to witness the presence of peritoneal tumors). (**C**,**D**) NOD/SCID mice were injected subcutaneously with A2780-Ctrl (*n* = 5), A2780-145 (*n* = 6), SKOV3-Ctrl (*n* = 5) or SKOV3-145 (*n* = 6) cells. Mice injected with A2780 cells exhibited tumor volumes ranging from 20–200 mm^3^ at 8–12 days post-cell injection, while at day 19 (when the assays were stopped), exhibited tumor volumes greater than 1000 mm^3^. Mice injected with SKOV3 cells exhibited tumor volumes greater than 500 mm^3^ at 35–40 days post-cell injection, and the assays were stopped. * = *p* < 0.05 (Tukey’s test). (**E**) Summary table with in vivo results obtained post-euthanasia of animals, showing the main findings of the carcinomatosis assays. (**F**) Quantification of A2780 cells/mL in the ascitic fluid present in the animals (*** = *p* < 0.001 Tukey’s test). (**G**) Representative photographs of mesenteric tumor growth in the intraperitoneal cavity of mice injected with SKOV3 cells (scale in cm). Results are expressed as mean ± standard deviation.

To test the anti-tumoral properties of miR-145 in vivo, a non-obese diabetic/severe combined immune deficiency NOD/SCID female mouse model was used, with the injection of A2780 and SKOV3 cells previously transduced with two lentiviral constructs encoding miR-145, as described in the methodology section (Supplementary Figures S1 and S2). The natural course of EOC development using these cells is described in Supplementary Figure S3. Mice with EOC xenografts that over-expressed miR-145 (145) retarded tumor formation in subcutaneous lateral flank, compared with the control EOC group (Ctrl) (*p* < 0.05). Importantly, tumor volume in control mice injected with A2780-Ctrl cells was at least five times greater than that of tumors developed with A2780-145 cells at 19 days (Figure 4A–D). A similar effect was observed in EOC xenografts using SKOV3 cells: tumor volume formed by SKOV3-Ctrl cells was at least three times greater than the xenografts from mice injected with SKOV3-145 cells (*p* < 0.05; Figure 4C,D).

According to the analysis of metastatic behavior, over-expression of miR-145, both in A2780 and SKOV3 cells, strongly decreased the presence of malignant ascites and diaphragmatic metastasis (Figure 4E). In agreement with these results, over-expression of miR-145 in metastatic cells (SKOV3) diminished mesenteric tumor formation, compared with SKOV3-Ctrl cells (Figure 4E–G). These results suggest that the increase in miR-145 levels in EOC xenografts exerts anti-tumoral effects and that miR-145 is a metastatic suppressor, both in vitro and in vivo.

### 2.5. miR-145 Regulates c-MYC and VEGF Protein Levels in EOC Cells

**Figure 5:**
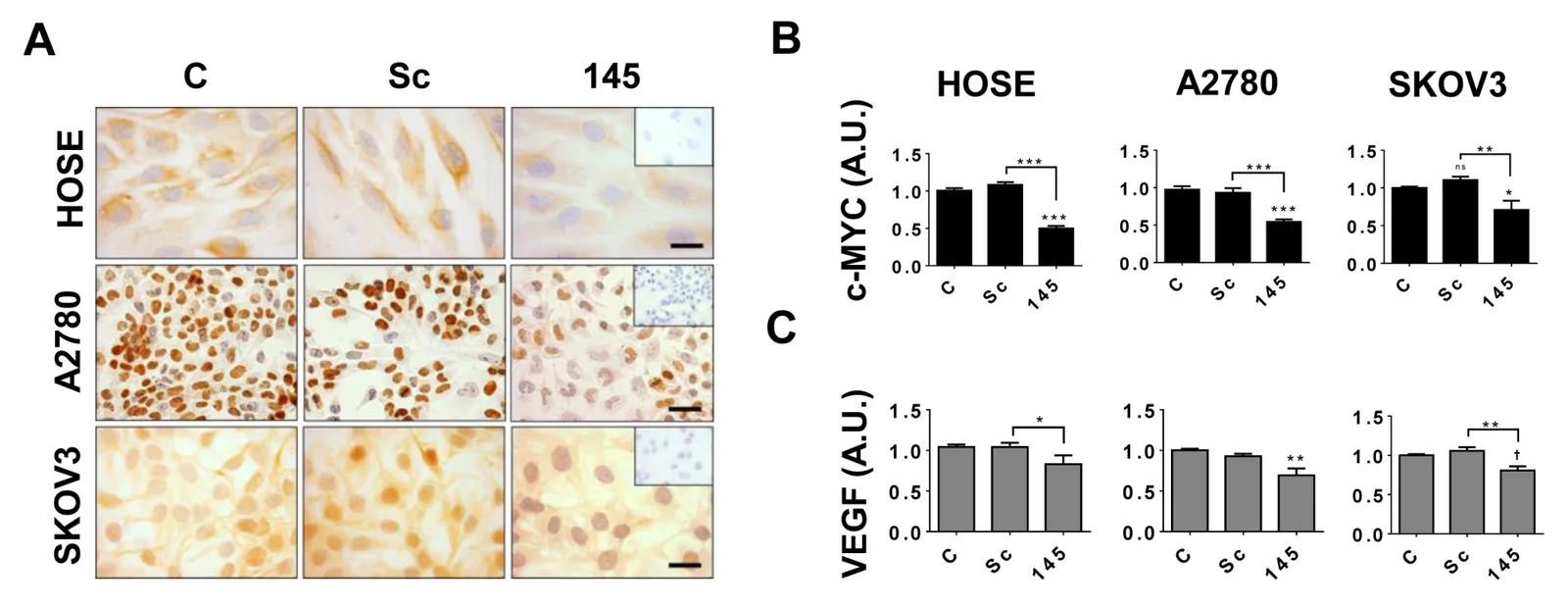
Effect of miR-145 upregulation on c-MYC and vascular endothelial growth factor (VEGF) levels of ovarian cells. Ovarian cells were transfected with miR-145 (145), a scrambled sequence (Sc) or none (C, control), using Lipofectamine 2000. Then, c-MYC levels were assessed by immunohistochemistry and VEGF levels were quantified by enzyme-linked immunosorbent assay (ELISA). (**A**) Representative images of c-MYC immunodetection (brown) in ovarian cells, under the respective conditions. Harris´ Hematoxylin (blue) was used as a counterstain. Right corner: negative control (without primary antibody). Bar = 50 µm. (**B**) Analysis of c-MYC immunodetection in ovarian cells. *N* = 4 (4–10 pictures per condition were analyzed). (**C**) VEGF levels in culture supernatants of ovarian cells determined by ELISA. *N* = 4. * = *p* < 0.05, ** = *p* < 0.01 and *** = *p* < 0.001 (Kruskal–Wallis test and Dunn’s post-test), respect to the control condition, or as indicated. Ns = statistically non-significant. † = *p* < 0.01, compared with the control condition (Mann–Whitney test).

Cell proliferation and angiogenesis are relevant processes during EOC progression. Therefore, we studied two important proliferative and angiogenic proteins, c-MYC and VEGF, which are downstream targets of NGF/TRKA signaling [9,12,15]. The current results show that miR-145 over-expression decreased c-MYC protein levels in all ovarian cell lines (*p* < 0.01 for HOSE and A2780 cells, *p* < 0.05 for SKOV3 cells; Figure 5A,B), and also decreased VEGF levels in culture supernatants of all ovarian cell lines (*p* < 0.05 for HOSE and SKOV3 cells, *p* < 0.01 for A2780 cells; Figure 5C).

### 2.6. In Silico Analysis of miR-145 and NGF/TRKA Targets

Since the upregulation of miR-145 decreases cell proliferation and several oncoproteins are regulated by NGF/TRKA (VEGF, c-MYC), we decided to study whether NGF/TRKA could regulate miR-145 levels. For the first step, an in silico analysis was performed (Table 1), which suggests that miR-145 may upregulate several proteins downstream of NGF/TRKA signaling.

### 2.7. NGF Decreases miR-145 in Ovarian Cells

**Figure 6:**
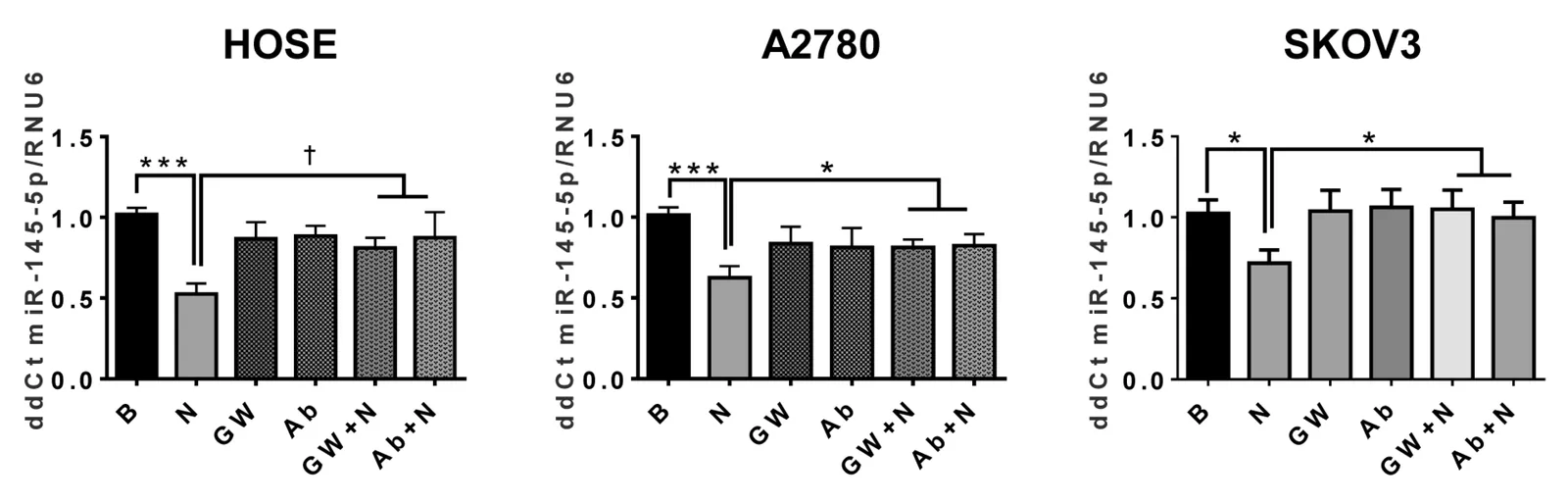
NGF decreases miR-145 levels in epithelial ovarian cells. miR-145 levels were determined by qRT-PCR in ovarian cell lines under the following treatments: Without stimuli (B, basal condition), NGF (N, 100 or 150 ng/mL for HOSE/A2780 and SKOV3, respectively), GW441756 20 nM (GW, specific TRKA inhibitor) or a neutralizing antibody against NGF (Ab, 5 μg/mL). *N* = 5 or more independent experiments. * = *p* < 0.05 and *** = *p* < 0.001, as indicated according to the Kruskal–Wallis test and Dunn’s post-test. † = *p* < 0.05 (Mann–Whitney test, as indicated). Results are expressed as standard error of mean (SEM).

Since the expression of NGF and its high-affinity receptor TRKA increase during EOC progression and they are considered as potential markers of this disease [9], we studied the possible association between NGF/TRKA and miR-145. The results show that NGF stimulation decreased miR-145 levels in all ovarian cell lines studied (*p* < 0.01 for HOSE and A2780 cells, *p* < 0.05 for SKOV3 cells; Figure 6). To elucidate whether these changes were specific and depended on the TRKA receptor, we used a neutralizing antibody against NGF (Ab) and a specific pharmacologic inhibitor of the TRKA receptor (GW). The results show that these inhibitors prevented the NGF-dependent decrease in miR-145 levels in the three cell lines (*p* < 0.05; Figure 6). These results indicate that NGF stimulation decreases miR-145 levels in ovarian cell lines in a TRKA-dependent manner.

### 2.8. NGF Stimulation Decreases Transcription of miR-145 in EOC Cells

**Figure 7:**
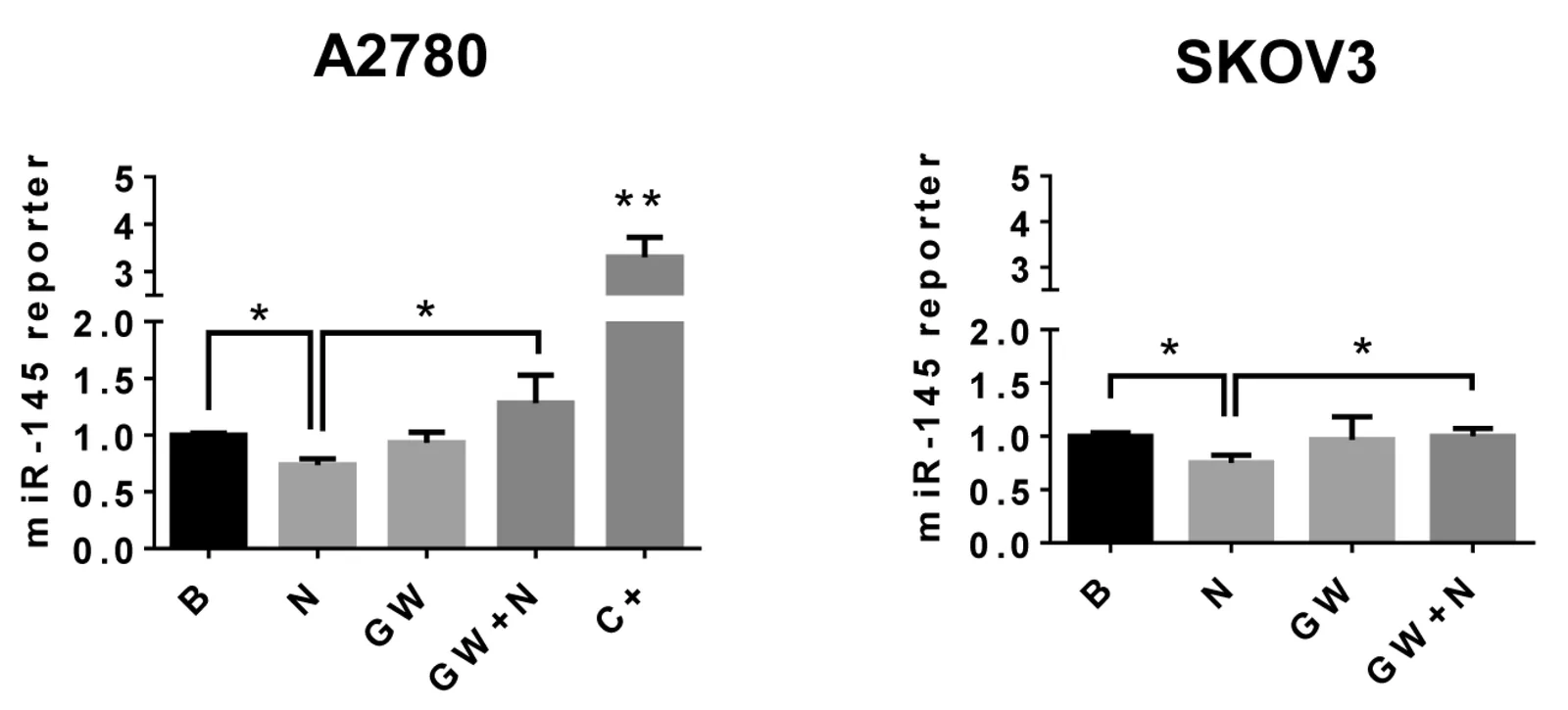
NGF inhibits the transcription of miR-145 in EOC cells. EOC cells were stimulated with NGF (N, 100 or 150 ng/mL for A2780 and SKOV3, respectively) and treated with the specific TRKA inhibitor GW441756 (GW, 20 nM). Then, gene-reporter assays were performed to evaluate miR-145 transcription. B: basal condition (without stimuli). C+: positive control (hydrogen peroxide 300 µM). * = *p* < 0.05 and ** = *p* < 0.01, as indicated (Kruskal–Wallis test and Dunn’s post-test). Results are expressed as standard error of mean (SEM).

In order to evaluate whether the decrease in miR-145 induced by NGF stimulation in EOC cells was associated with a decrease in miR-145 transcription, we performed reporter assays in EOC cells transfected with a luciferase reporter plasmid containing the miR-145 promoter. As shown in Figure 7, NGF stimulation decreased the transcriptional activity of the miR-145 promoter in both A2780 and SKOV3 cells (*p* < 0.05). Additionally, the use of the specific TRKA inhibitor (GW) prevented the NGF-mediated decrease in miR-145 promoter activity in EOC cells.

### 2.9. Overexpression of miR-145 Blocks the NGF-Mediated Effects in Epithelial Ovarian Cells

**Figure 8:**
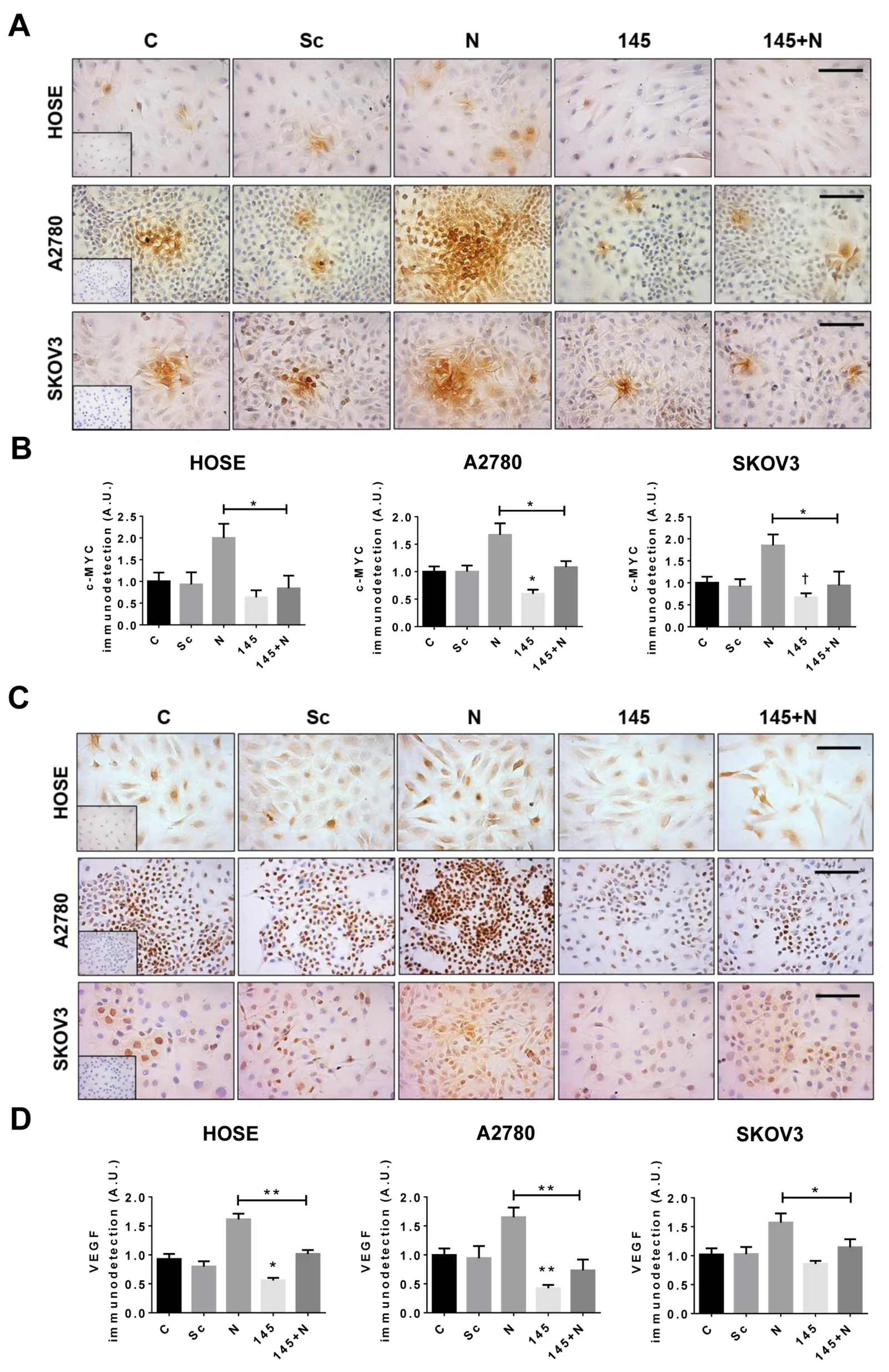
miR-145 overexpression blocks the NGF-mediated increase in c-MYC and VEGF in EOC cells. EOC cells were transfected with miR-145 (30 nM, 48 h) and stimulated with NGF for 3 h (100 or 150 ng/mL for HOSE/A2780 and SKOV3, respectively). (**A**,**C**) representative pictures of VEGF and c-MYC immunohistochemistry. Left insert: negative control (cells without primary antibody). Bar = 20 µm. (**B**,**D**) semi-quantification of VEGF and c-MYC immunodetection. C: control condition (cells treated with only lipofectamine). SC: cells transfected with the scrambled sequence. N: cells treated with lipofectamine and NGF, 145: cells transfected with miR145. * = *p* < 0.05 and ** = *p* < 0.01, as indicated (Kruskal–Wallis test and Dunn’s post-test). † = *p* < 0.05 (Mann–Whitney test), compared to the basal condition. Results are expressed as standard error of mean (SEM).

In order to find out if miR-145 could inhibit the NGF-mediated increase in oncoproteins, EOC were transfected with miR-145 and stimulated with NGF. As shown in Figure 8, NGF stimulation increased the presence of c-MYC and VEGF in HOSE, A2780 and SKOV3 cells. However, previous transfection of ovarian cells with miR-145 prevented the increase in these proteins dependent on NGF (*p* < 0.05 and *p* < 0.01; Figure 8B,D and Supplementary Figure S4).

## 3. Discussion

NGF/TRKA are important molecules involved in the pathogenesis and progression of EOC [9] and their contribution to miR regulation has been poorly studied. Here, we described for the first time, the relationship between NGF/TRKA and miR-145 in the context of cancer, contributing to clarify an additional pro-tumoral mechanisms of NGF/TRKA in EOC cells, which involves miR-145 regulation. Our results showed that miR-145-5p levels diminish during EOC progression. Moreover, NGF contributes to the decrease in miR-145-5p levels in EOC in a TRKA-dependent manner, which involves a reduction in the transcriptional activation of the miR-145 promoter. On the other hand, the over-expression of miR-145 in EOC mouse xenografts showed tumor suppressor effects, because it decreased tumor formation and suppressed metastasis behavior. Our results confirm previous findings of other researchers [39,42,43] and contribute to understanding the role of miR-145 in EOC pathogenesis, which regulates important oncoproteins, such as c-MYC and VEGF, which are increased by NGF/TRKA signaling [9,15]. Since miR-145 is naturally present in cells, restoring levels of miR-145 in EOC could be considered as a new therapeutic strategy.

miRs are endogenous, small non-coding RNAs that regulate gene translation [24]. miRs bind to mRNAs and block their lecture or induce mRNA degradation, which produces a decrease in the protein translation of targeted messengers [22]. Reports have indicated that, in cancer cells, miRs are deregulated; tumor-suppressor miRs (which control oncoproteins) are decreased and onco-miRs (which regulate tumor-suppressor proteins) are increased. This affects important hallmarks of cancer, including the stimulation of proliferative signaling, the evasion of growth suppressors, resistance to cell death, the activation of invasion and metastasis, and the induction of angiogenesis [25,26,27,47]. Importantly, one miR could regulate thousands of mRNAs and proteins [23,24]. Therefore, miRs constitute a new suitable therapeutic strategy in the cancer scenario, for instance, by re-establishing endogenous levels of tumor-suppressor miRs that are downregulated in EOC cells. Since miRs are endogenous RNAs, side effects or complications could be milder, and this strengthens the idea of using miRs as a new therapy or adjuvant therapy for cancer.

Studies have reported that miR-145 is downregulated in several models of cancers, such as gastric cancer [48], breast cancer [49], colorectal cancer [50] and esophageal squamous cell carcinoma [51], among others. In particular, in ovarian cancer, our results are consistent with other reports, which have shown that miR-145 levels in ovarian cancer tissues are lower than in non-tumoral tissues [39,41,52,53,54]. Additionally, and also in agreement with the present results, several reports have shown in in vitro models of ovarian cancer cells that over-expression of miR-145 decreases different tumor proprieties, such as cell proliferation, invasion, colony formation [39] and epithelial–mesenchymal transition [55], or increases sensibility to chemotherapy [54]. Here, we observed that the upregulation of miR-145 in EOC cells not only decreases tumoral proprieties in vitro, but also decreases important parameters in vivo, such as tumor formation, tumor size, the number of mesenteric tumor nodules in a carcinomatosis model and the presence of ascites in mice. Many women with advanced EOC have ascites, which indicates that cancer cells have spread outside the ovary. Because cell dissemination of EOC is a key point in EOC progression and over 90% of mortality from cancer can be attributed to metastases [56,57], an interesting projection is the development of miR-145-based therapy, which could help slow down disease progression when complemented by chemotherapy. This idea is based on an antisense therapy that has been recently approved by the Food and Drug Agency (FDA) and shown interesting results: Mipomersen, which binds to the mRNA encoding apolipoprotein B-100 [58].

Differences in miR expression have not only been described in tissue biopsies, but also in blood samples of cancer patients [48,59]. As in tissues, some miRs have been proposed as potential blood biomarkers for cancer diagnosis and prognosis [60,61,62,63]. Previous studies have proposed using miR-145 as a biomarker of ovarian cancer because miR-145 serum levels are significantly down-regulated in patients with ovarian cancer and benign ovarian tumors, compared to healthy controls [38,64]. Furthermore, patients with low miR-145 serum levels have a significantly shorter median overall survival rate [38], which corroborates the antitumor effects of miR-145 described in the present work.

Several reports have shown that miR expression is deregulated in human cancers through various mechanisms, including amplification or deletion of miRNA genes, abnormal transcriptional control of miRNAs and deregulated epigenetic changes and defects in the miRNA biogenesis machinery [65]. Some reports have suggested that neurotrophins such as brain-derived neurotrophic factor (BDNF) could regulate miR abundance through at least three mechanisms: (1) ERK/AKT-dependent modulation of endoribonuclease Dicer, producing an upregulation of onco-miR synthesis; (2) ERK-dependent regulation of the RNA-binding protein Lin28a, which blocks selected miR biosynthesis and (3) transcriptional regulation of miR expression through the activation of transcription factors such as cyclic adenosine monophosphate response element-binding (CREB) and nuclear factor kappa-light-chain-enhancer of activated B cells (NF-κB) [66,67,68]. Because NGF/TRKA produces the activation of similar signaling pathways as those of BDNF and our findings showed that NGF/TRKA decreased miR-145 levels in EOC cells, the possible mechanism of this decrease could be mediated by the last two alternatives: ERK-dependent Dicer modulation or modulation of miR-145 transcription through CREB, the main mediator of neuronal neurotrophic responses [69]. CREB activation has been reported by NGF stimulation in neurons [70,71] and cancer cells [72,73]. Therefore, this interesting aspect should be addressed more deeply in the future.

Another relevant point is that miR-145 could be considered as a possible complementary therapy in EOC, since miR-145 regulates several important proteins in the context of drug resistance, such as ABCB1 efflux transporters (MDR1 or P-glycoprotein) in intestinal epithelial cells [74] or ABC in gallbladder cancer cells [75]. Evidence has indicated that overexpression of miR-145 sensitizes gallbladder cancer cell lines to cisplatin by increased ABCC1 expression. Low levels of ABCC1 in gallbladder cancer predicts poor prognosis of patients who received chemotherapy [75]. Since the first-line chemotherapy for EOC includes platinum, these antecedents support the idea of considering miR-145 as a potential new co-adjuvant therapy in the context of EOC by increasing platinum sensibility.

A possible limitation of this work is that the ovarian cell lines A2780 and SKOV3 are not representative of high grade serous (HGS) ovarian carcinoma [76], the most aggressive form of the disease. However, a recent report showed that non-HGS EOC cell lines migrate and invade to a greater extent than those derived from HGS carcinomas [77], suggesting that non-HGS EOC have a higher metastatic potential than cells derived from HGS carcinomas. Our results showed that miR-145 upregulation decreases the invasion and migration potential of A2780 and SKOV cells, and decreases the metastatic potential of these cells in an in vivo model, suggesting that a possible therapy based on miR-145 could be promissory for the prevention of cancer dissemination.

**Figure 9:**
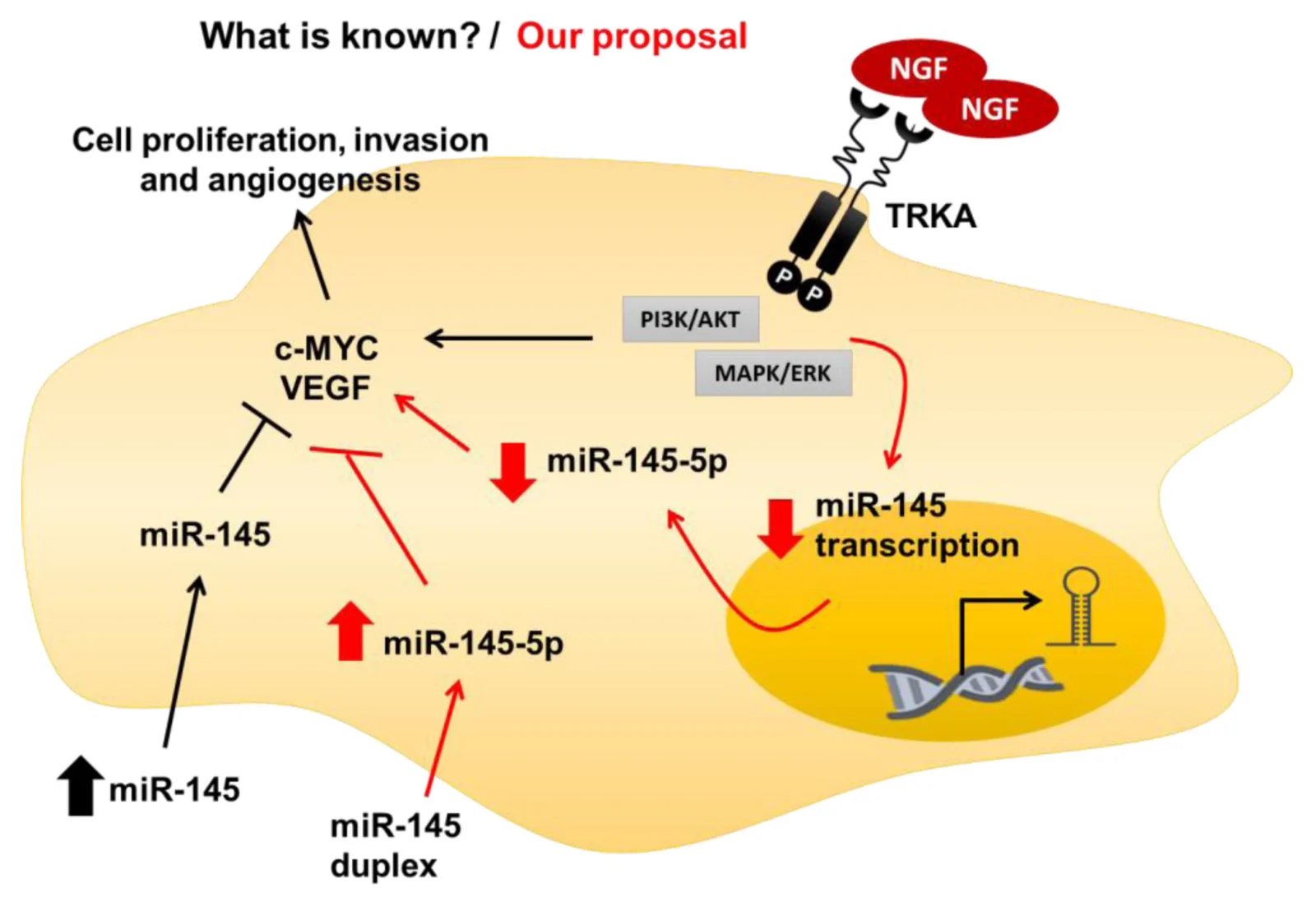
State of the art about miR-145 in EOC and our findings in the current study. Since miR-145 regulates important oncoproteins involved in NGF/TRKA signaling, such as VEGF and c-MYC, we decided to study whether NGF stimulation or TRKA inhibition modulates miR-145 levels, particularly miR-145-5p. According to our results, NGF/TRKA decreases miR-145 transcription and miR-145-5p abundance. Because miR-145 decreases protein levels of c-MYC and VEGF, our results suggest that at least in part, the pro-tumoral effects of the NGF/TRKA system could be mediated by the decrease in miR-154-5p in EOC cells.

In summary, the present work describes a novel pro-tumoral mechanism of NGF/TRKA in EOC cells through the decrease in miR-145 transcriptional activity and abundance in EOC cells. In addition, we replicated experiments performed in previous reports that showed that miR-145 regulates important processes in cancer cells, such as cell proliferation, migration and invasion, by controlling c-MYC and VEGF protein levels (Figure 9). The over-expression of miR-145 in EOC cells resulted in tumor suppressor effects, such as decreased subcutaneous tumor formation and suppressed metastasis behavior of EOC cells. Since miR-145 is naturally present in cells, these results support that a miR-145-based therapy could be considered as a new therapeutic strategy in EOC, through the development of formulations for co-adjuvant therapy to chemotherapy, intended to decrease ascites and improve the life quality of patients.

## 4. Material and Methods

### 4.1. Tissue Collection

Tissue samples were obtained from patients in two institutions from Santiago, Chile: Hospital Clínico Universidad de Chile and the National Institute of Cancer. An expert pathologist classified the ovarian samples as: inactive ovaries from post-menopausal women (IOV), cystadenomas or borderline tumors (Tum) and serous epithelial ovarian cancer (EOC). Each patient signed an informed consent form, approved by the local Ethical Committee (Record N°022, 2016). After surgery, tissues were immediately frozen in liquid nitrogen and kept frozen until used for miR extraction.

### 4.2. Cell Lines

The ovarian cell line human ovarian surface epithelial cells (HOSE, from a menopausal woman, immortalized by SV40-Tag) was donated by Dr. Davie Munroe (NCI, National Institutes of Health) [44]. The A2780 cell line (human epithelial ovarian cancer) was obtained from the European Collection of Authenticated Cell Cultures (ECACC, Porton Down, UK) and the SKOV-3 cell line was obtained from the American Type Culture Collection (ATCC, Manassas, VA, USA). All cell lines were regularly tested for mycoplasma. HOSE and A2780 cells were cultured in Dulbecco’s modified medium (DMEM) Ham F12 without phenol red, supplemented with 10% fetal bovine serum (FBS) at 37 °C with 5% CO_2_ until reaching 80% confluence. SKOV-3 cells were cultured with Roswell Park Memorial Institute (RPMI) 1640 medium supplemented with 10% fetal bovine serum (FBS) at 37 °C with 5% CO_2_ until reaching 80% confluence. Cells were serum-deprived for 24 h and stimulated with NGF 100 or 150 ng/mL (Sigma-Aldrich Co. St Louis, MO, USA) and treated with the specific TRKA inhibitor GW441756 (20 nM; Tocris, Bristol, UK) or a neutralizing antibody against NGF (5 μg/mL; Abcam 6199, Cambridge, UK) for 3 h. Inhibitors were added 1 h before NGF stimulation.

### 4.3. miR Extraction, RT-PCR and qPCR

Extraction of miRs was performed using QIAzol and miRNeasy mini kit (Qiagen, Hilden, Germany), according to the manufacturer’s instructions. Reverse transcription of RNA was performed with miScript II RT kit (Qiagen). Later, quantitative real time PCR was performed using the miScript SYBR Green qPCR kit (Qiagen) in a StepOne Real-Time PCR thermocycler (Applied Biosystems, Foster City, CA, USA). The results were calculated using the ΔΔCq method [78]. Primers and PCR programs are described in Supplementary Table S1. U6 small nuclear RNA (RNU6) was used as housekeeping miR and bidistilled water was used instead of cDNA as a negative control.

### 4.4. Transfection

HOSE, A2780 and SKOV-3 cell lines were transfected with synthetic miR-145 (Integrated DNA Technologies, Coralville, IA, USA) or a scrambled sequence (37007, Santa Cruz Biotechnology, Dallas, TX, USA) using Lipofectamine 2000 in a final concentration of 10 µM (Invitrogen, Carlsbad, CA, USA), previously diluted in culture medium, according to the manufacturer’s instructions (1/100). 500,000 cells were seeded in 6-well plates and transfected with 0, 30 and 60 nM of miR-145 for 48 h in 1 mL of DMEM-Ham F12 or RPMI 1640 medium supplemented with 2% FBS. Transfection efficiency was evaluated by real-time PCR and fluorescence microscopy, using the internal Cy5 labelling (red-fluorescent dye) in the miR-145 duplex. Based on the first results, we decided to perform all experiments with miR-145 30 nM.

### 4.5. Gen Reporter Assay for miR-145 

Transcriptional activation of the miR-145 promoter was evaluated by the firefly luciferase (FLuc) reporter assay, as previously described [79]. Cells (450,000) were transfected using Lipofectamine 2000 (Invitrogen) in a final concentration of 15 µM (A2780) or 20 µM (SKOV3), with 500 ng of the plasmid P-del (pTransluc-miR-145-2), which was a gift from Kenneth Kosik (Addgene plasmid #21500; http://n2t.net/addgene:21500; RRID:Addgene_21500) and 500 ng (A2780) or 1000 ng (SKOV3) of pON-FLASH (beta-galactosidase transfection control) [80]. Cells were serum-deprived for 2 h and stimulated with GW441756 20 nM and NGF 100 ng/mL (A2780 cells) or 150 ng/mL (SKOV3 cells) for 24 h and then, the cells were lysed and FLuc activity was measured with the dual luciferase reporter assay system (Promega, Madison, WI, USA), according to the manufacturer’s instructions. β-galactosidase activity was measured as previously described [80]. Hydrogen peroxide 300 µM was used as positive control of miR-145 transcriptional activation [81].

### 4.6. Immunocytochemistry

HOSE, A2780 and SKOV-3 cells were seeded in 24-well plates (50,000 cells/well) containing 12 mm round coverslips and were transfected for 48 h, as described in the transfection section, with miR-145 or a scrambled sequence in 500 µL of DMEM-Ham F12 or RPMI 1640 medium supplemented with 2% FBS. Then, cells were fixed with 4% paraformaldehyde in phosphate-buffered saline solution (PBS) and permeabilized with 0.3% Triton X-100. Endogen peroxidases were blocked with 3% hydrogen peroxide in PBS and non-specific binding was blocked using 2% of FBS in phosphate-buffered saline solution–bovine serum albumin (PBS–BSA). Samples were incubated overnight with the following primary antibodies: rabbit monoclonal anti-human c-MYC 1:500 (Cell signaling Technology #5605, Danvers, CO, USA), mouse monoclonal anti-VEGF 1:500 (Abcam, ab1316) and mouse monoclonal anti-human Ki-67 1:250 (#23900, Santa Cruz Biotechnology). The secondary antibodies were Goat anti-mouse and Goat anti-rabbit IgG (#115035 and #111035, Jackson ImmunoResearch, West Grove, PA, USA) and were used 1:300 for 1 h at 37 °C. Color was developed using 3,3′-diaminobenzidine (DAB) (DakoCytomation, Carpinteria, CA, USA) as a substrate. Slides were evaluated using an optic Olympus BX51 microscope (Olympus Corporation, Tokyo, Japan) with a Micro-Publisher 3.3 RTV camera (Q Imaging, Surrey, BC, Canada). Immunodetection was evaluated by obtaining the mean of integrated optical density (IOD) of 4–10 pictures per condition, with the Image Pro Plus 6.2 computer software (Media Cybernetics Inc., Silver Spring, MD, USA). Uneven illumination was corrected using a control image, as described previously [82].

### 4.7. MTS Assay

Using 96-well plates, 5000 cells were seeded per well and transfected with miR-145 as described in the transfection section. Then, the MTS assay (#211091 Abcam) was performed according to the manufacturer’s instructions.

### 4.8. Enzyme-Linked Immunosorbent Assay (ELISA) for VEGF

VEGF levels in culture medium were measured using an enzyme-linked immunosorbent assay (ELISA) kit (DVE00 R&D Systems, Minneapolis, MN, USA), according to the manufacturer’s instructions. The inter-assay variation coefficient was 6.7% and assay sensitivity was 5.0 pg/mL.

### 4.9. Migration Assay

EOC cells (50,000) were transfected as described in the transfection section and were added to the upper chamber of 6.5 mm transwells with 8.0 µm pore polycarbonate membrane inserts (Corning Inc., Corning, NY, USA) coated on the lower surface with fibronectin (Gibco, Thermo Fisher Scientific, Waltham, MA, USA). Ovarian cancer cells were allowed to migrate at 37 °C for 4 h (SKOV3) or 8 h (A2780). Cells were then stained overnight with crystal violet. Cells attached to the lower membrane surface were counted. Inserts were photographed (3–5 pictures for each experimental condition with 200× magnification) and analyzed using Fiji Image J (developed by the National Institutes of Health/University of Wisconsin, Madison, WI, USA).

### 4.10. Invasion Assay

BioCoat matrigel invasion chambers (Corning Inc. #354480, Corning, NY, USA) were hydrated overnight at 4 °C and 30,000 transfected cells (A2780 and SKOV-3) were re-suspended in DMEM-Ham F12 medium without FBS and were added to the upper chamber. The inserts were incubated at 37 °C for 24 h (A2780 cells) or 16 h (SKOV3 cells) and then cells were fixed with 500 µL of methanol (−20 °C) for 2 min and stained with 500 µL of 1% Toluidine blue. Cells attached to the lower membrane surface were counted. Inserts were photographed (3–5 pictures for each experimental condition with 200× magnification) and analyzed using Fiji Image J.

### 4.11. Over-Expression of miR-145 in EOC Cells by Transduction with Lentiviral Particles

For miR over-expression assays we used the pGPGLenti-human cytomegalovirus-green fluorescent protein (CMV-GFP)-puro vector, previously validated for microRNA expression [83]. After amplification of the primiR-145 sequence (300 pb flanking the miR-145 stemloop: MI0000461), this insert was cloned in the SalI site in forward sense (pGPG-145). As a control, we also used the empty vector (pGPG-Ctrl). Each construct was co-transfected with the helper plasmids (pPAX2 and pVSV-G) in HEK 293T cells. Supernatants with lentiviral particles were harvested 48 h post-transfection and the cell debris was removed by centrifugation.

Prior to transduction with lentiviral particles, EOC cells were treated with medium including 6 µg/mL of Polybrene (Hexadimethrine bromide H9268, Sigma-Aldrich) for 30 min. Then, the medium was removed and the lentiviral particles were added (ratio 1:1 in medium) to obtain A2780 and SKOV3 cells that overexpressed miR-145 (A2780/SKOV3-145) and cells with the empty vector (A2780/SKOV3-Ctrl). After 18 h, medium was changed again until harvesting for analysis 72 h after transduction.

The GFP-expressing cells (all the transduced cells) were sorted using a GFP mark in a BD FACS Canto A equipment (BD Biosciences, San Jose, CA, USA) (Supplementary Figures S1 and S2).

### 4.12. Mouse Xenografts of EOC Cells with Stable miR-145 Over-Expression

Animal studies were conducted in accordance with appropriate guidelines of the Ethical Committee of Universidad del Desarrollo. Immune-compromised NOD/SCID mice were obtained from Jackson Laboratories (Bar Harbor, ME, USA) and maintained at the animal facility of Universidad del Desarrollo under specific pathogen-free conditions (High Efficiency Particulate Air (HEPA) filter system room), in a temperature-controlled environment with a 12/12 h light/dark schedule, sterile food and water ad libitum.

### 4.13. Subcutaneous Tumor Formation and Peritoneal Carcinomatosis Assay

Tumor formation and metastasis in the intraperitoneal cavity was evaluated in immune-compromised mice using two EOC cell lines (A2780 and SCOV3). In each case, mice were injected with 20 × 10^7^ or 8 × 10^6^ cells, respectively, in 250 µl of saline solution (intraperitoneal cavity) or in 100 µL of saline solution/matrigel (1:1) (subcutaneous lateral flank). Intraperitoneal behavior was evaluated by measuring the abdominal volume. The appearance of tumors in the flank of mice was monitored by palpitation, the diameters of the resulting tumors were periodically measured and tumor volumes were calculated using the formula width^2^ × length × π/6 [84]. Subcutaneous tumor formation was considered as a positive control of tumor formation during the carcinomatosis intraperitoneal assays. 

Two groups (5 and 6 NOD/SCID mice) were inoculated with control A2780 EOC cells (A2780-Ctrl) and with EOC cells that over-expressed miR-145 (A2780-145), respectively. Animals were euthanized on day 19 post cell injection. On the other hand, two groups (5 and 6 NOD/SCID mice) were inoculated with control SKOV3 EOC cells (SKOV3-Ctrl) and SKOV3 cells that over-expressed miR-145 (SKOV3-145), respectively. Animals were euthanized on day 40 post cell injections.

After the animals were euthanized, the mesentery tissue and retroperitoneal tumor mass were excised and fixed in 4% paraformaldehyde. Malignant ascites were collected, and total cell number was determined by trypan blue exclusion assay. The final in vivo evaluation was performed in a double-blinded fashion.

### 4.14. In Silico Prediction of miR-145: Target Interactions

Bioinformatic analysis was conducted using the MIENTURNET tool [85], which includes data from TargetScan (predictive) and miRTarBase (experimentally validated) to perform interaction analysis. In addition, the miRWalk tool [86] was used to perform predictive interaction analysis by its own algorithm. It includes data from TargetScan, miRTarBase and miRDB databases. MIENTURNET analysis settings were the following: (1) Minimum number of miRNA-target interactions: 1; (2) *p*-value: < 0.05 and (3) threshold adjusted *p*-value (false discovery rate): 0.2. All other parameters were set at default. miRWalk analysis was performed using a score filter >0.95 (being 1 the maximum score indicating maximum prediction). Only predicted interactions with the 3′UTR region was assessed.

### 4.15. Statistical Analysis

For in vitro and ex vivo results, differences between groups were analyzed using Kruskal–Wallis and Dunn’s post-test. Results were considered significant with a *p* < 0.05 and they were expressed as mean ± standard error of the mean (SEM). For in vivo experiments, results were compared using the multiple comparison test and Tukey’s post-test (IC 95%). Results were expressed as mean ± standard deviation (SD).

## Figures and Tables

**Table 1:** In silico analysis of miR-145 and nerve growth factor (NGF)/TRKA targets. The predicted information was evaluated using MIENTURNET and miRWalk online tools.

miR-145-3p/5p	Target Gene	Protein Affected (Downregulated)	Tumorigenic/ Metastatic Effect	NGF-Mediated Effect
miR-145-3p/5p	MYC *	c-MYC	Transcription of proliferation genes	Upregulation
miR-145-3p/5p	*MYCBP2*	MYC Binding Protein 2	Transcription of proliferation genes	Upregulation
miR-145-3p/5p	VEGFA *	Vascular Endothelial Growth Factor	Tumor angiogenesis	Upregulation
miR-145-5p	*ADAM17*	ADAM metallopeptidase domain 17	ECM remodeling	Upregulation
miR-145-5p	*AKT1*	AKT Serine/Threonine Kinase 1	PI3K/Akt signaling pathway	Upregulation
miR-145-3p	*PIK3C2A*	Phosphoinositide-3-kinase, class 2, alpha polypeptide	PI3K/Akt signaling pathway	Upregulation
miR-145-5p	*CAV1*	Caveolin 1	Cell migration/invasion	Upregulation
miR-145-5p	*NGF*	Nerve Growth Factor	NGF signaling pathway	-
miR-145-5p	*NTRK1*	High-affinity Nerve Growth Factor Receptor (TRKA)	NGF signaling pathway	-
miR-145-5p	*BEX3*	Nerve Growth Factor Receptor Associated Protein 1	NGF signaling pathway Apoptosis regulation	Upregulation
miR-145-3p/5p	*MAPK1*	Mitogen-activated Protein kinase 1 (ERK)	Cell proliferation signaling	Upregulation
miR-145-3p/5p	*MYCT1*	MYC Target 1 (MYC target protein 1)	Cell proliferation signaling	Downregulation
miR-145-3p/5p	*MAPK10*	Mitogen-activated protein kinase 10 (JNK3)	Cell proliferation signaling	Downregulation
miR-145-3p/5p	*CDH5*	Cadherin-5	Cell adhesion	Downregulation
miR-145-3p/5p	*BCL2*	B-cell CLL/lymphoma 2	Anti-apoptosis Apoptosis regulation	Downregulation

* Experimentally validated Interaction between miR-145-5p with MYC and VEGFA.

## Supplementary Materials

The following are available online at https://www.mdpi.com/1422-0067/21/20/7657/s1.
