# Correction: Garrido et al. NGF/TRKA Decrease miR-145-5p Levels in Epithelial Ovarian Cancer Cells. *Int. J. Mol. Sci.* 2020, *21*, 7657

**DOI:** 10.3390/ijms27135687

**Published:** 2026-06-24

**Authors:** Maritza P. Garrido, Ignacio Torres, Alba Avila, Jonás Chnaiderman, Manuel Valenzuela-Valderrama, José Aramburo, Lorena Oróstica, Eduardo Durán-Jara, Lorena Lobos-Gonzalez, Carmen Romero

**Affiliations:** 1 Laboratorio de Endocrinología y Biología de la Reproducción, Hospital Clínico Universidad de Chile, Santiago 8380456, Chile; mgarrido@hcuch.cl (M.P.G.); ignacio.torres.p@ug.uchile.cl (I.T.); jose.aramburo@ug.uchile.cl (J.A.); 2 Departamento de Obstetricia y Ginecología, Facultad de Medicina, Universidad de Chile, Santiago 8380453, Chile; 3 Centro de Medicina Regenerativa, Facultad de Medicina, Clínica Alemana-Universidad del Desarrollo, Santiago 7710162, Chile; albaavila@gmail.com (A.A.); eduranj@udd.cl (E.D.-J.); 4 Programa de Virología, Instituto de Ciencias Biomédicas, Facultad de Medicina, Universidad de Chile, Santiago 8380453, Chile; jchnaiderman@med.uchile.cl; 5 Laboratorio de Microbiología Celular, Instituto de Investigación e Innovación en Salud, Facultad de Ciencias de la Salud, Universidad Central de Chile, Santiago 8320000, Chile; manuel.valenzuela@ucentral.cl; 6 Centro de Investigación Biomédica (CIB), Facultad de Medicina, Universidad Diego Portales, Santiago 8370007, Chile; lorenaorostica@gmail.com

In the original publication [1], there was a mistake in Figure 3C as published. Instead of including a representative photograph of each condition (C and SC), only photographs of condition C were incorporated in SKOV3 cells. The photographs used for the quantification of the experiments were taken with 400 X magnification, while the representative photographs of each condition (which were ultimately included in the paper) were taken after the performance and quantification of the experiments, with lower magnification (200 X) used for the better visualization of the results. The corrected version of Figure 3C appears below. The authors state that the scientific conclusions are unaffected. This correction was approved by the Academic Editor. The original publication has also been updated.

## Figures and Tables

**Figure 3:**
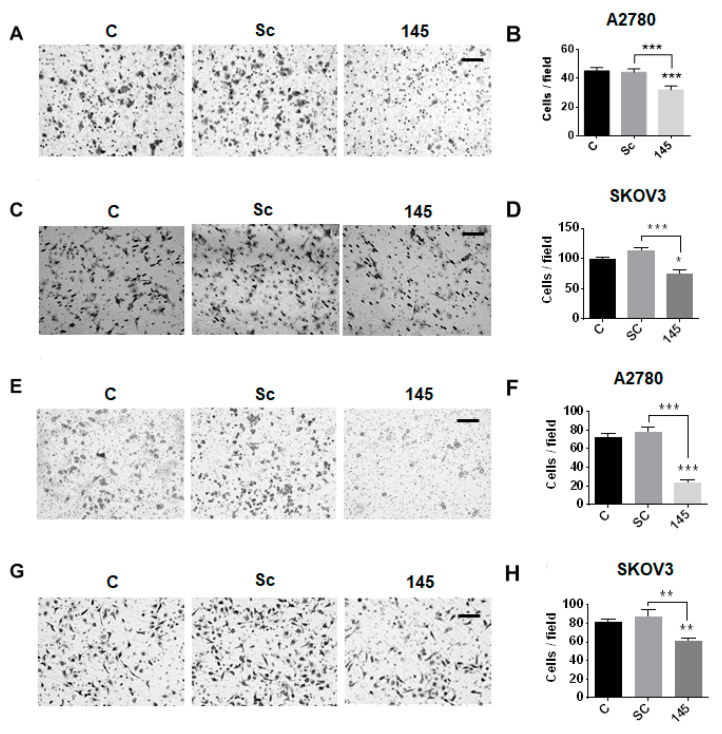
The over-expression of miR-145 decreases the migration and invasion ability of EOC cells. EOC cells were transfected with miR-145 (145), a scrambled sequence (Sc) or none (C, control), using Lipofectamine 2000 (levels of miR-145 after transfection are shown in Figure 2) and cell migration and invasion were assessed as described in the Methodology section. (**A**,**C**) Representative pictures of A2780 and SKOV3 cells after the migration assays (stain: crystal violet). (**B**,**D**) The quantification of the migration assays (cells that crossed the membrane/field) of the respective experimental groups. (**E**,**G**) Representative pictures of A2780 and SKOV3 cells after the invasion assays (stain: toluidine blue). Bar = 100 µm. (**F**,**H**) The quantification of the invasion assays (cells that crossed the membrane/field) of the respective experimental groups. *N* = 4 (3–5 pictures per condition were analyzed). Bar = 100 µm. * = *p* < 0.05, ** = *p* < 0.01 and *** = *p* < 0.001 (Kruskal–Wallis test and Dunn’s post-test), either with respect to the control condition, or as indicated. The results are expressed as standard error of mean (SEM).
