# Supplementary material for: NGF/TRKA Decrease miR-145-5p Levels in Epithelial Ovarian Cancer Cells
Source: Int J Mol Sci. 2020 Oct 16;21(20):7657. doi: 10.3390/ijms21207657 (PMC7589588; doi:10.3390/ijms21207657)
Supplement: Supplementary file 1 [file ijms-21-07657-s001.pdf]

Supplementary Materials

NGF/TRKA Decrease miR-145-5p Levels in Epithelial Ovarian Cancer Cells

Table S1. Primers and PCR program employed for miR detection.

	Primers	PCR Program T° and Time
miR-145-5p	miR-145_1 miScript Primer Assay (MS00003528, Qiagen)	94 °C 15 s<br>55 °C 30 s<br>70 °C 30 s<br>40 cycles
U6 small nuclear RNA (RNU6)	RNU6 miScript Primer Assay (MS00033740, Qiagen)	

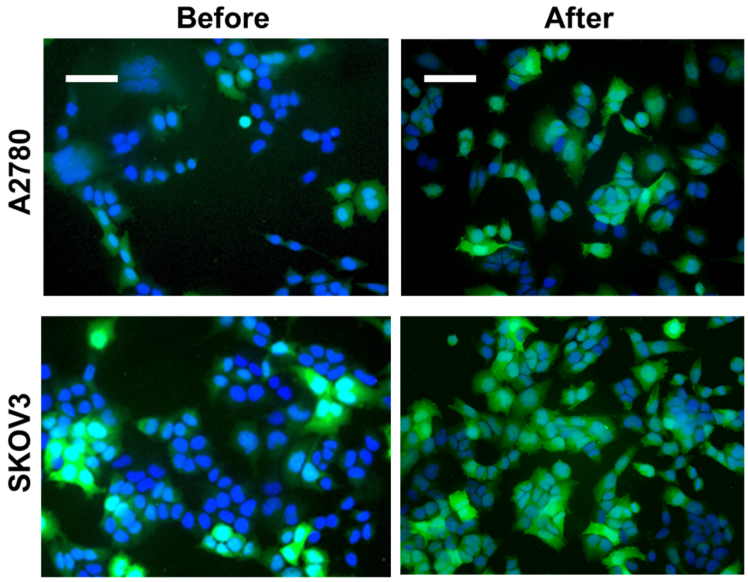

Figure S1. Ovarian cell lines (A2780 and SKOV3) were transduced according to the methodology section with the viral vector pGPG-145, which allows the expression of green fluorescent protein (GFP) and miR-145. Pictures show an enrichment of cells that express GFP (and therefore, miR-145) before and after cell sorting in a flow cytometer. Bar = 50 μm.

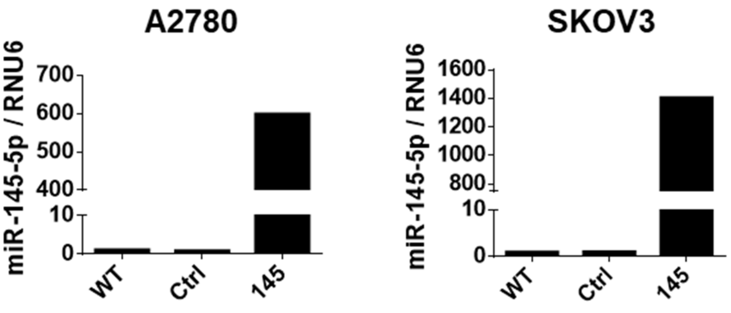

Figure S2. mi-R145 levels in EOC cells transduced with a lentiviral construct that overexpresses miR-145. EOC cell lines (A2780 and SKOV3) were transduced with two viral vectors and sorted in a flow cytometer, according to the methodology section. Then, miR-145 levels were measured by qRT-PCR (ΔΔCq method) in the same cells. WT = wild-type EOC cells. RNU6: U6 small nuclear RNA (housekeeping transcript).

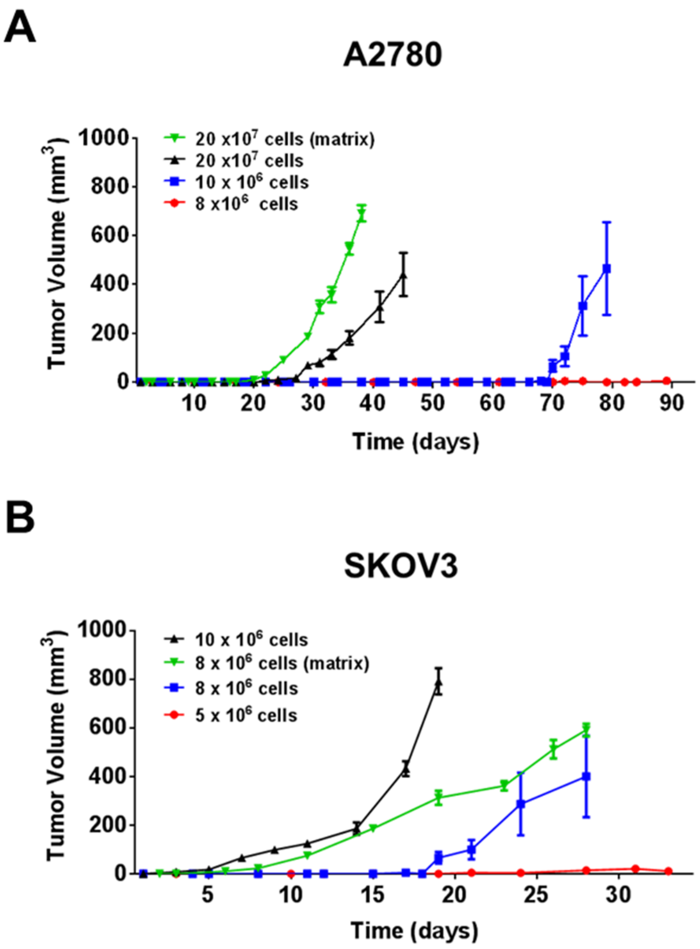

Figure S3. Time-course of subcutaneous tumour formation xenograft assays in NOD/SCID mice using A2780 and SKOV3 cell lines. Natural course of EOC xenografts using A2780 and SKOV3 cells. (**A**) A2780 cells (8×10^6, 10×10^6 and 20×10^7) were injected subcutaneously in 100 µL sterile saline solution and 20×10^7 cells were injected in 100 µL of saline solution/matrix 1:1 (Geltrex, Gibco-Thermo Fisher Scientific) into the left flanks of 16 NOD/SCID mice (n = 16 mice; 4 animals per group). (**B**) SKOV3 cells (5×10^6, 8×10^6 and 10×10^6) were injected subcutaneously in 100 µL sterile saline solution as described above and 8×10^6 cells were injected in 100 µL of saline solution/matrix 1:1 into the left flanks (n = 16 mice, 4 animals per group). Tumour growth was monitored every three days with a digital caliper and tumour volume (mm^3) was estimated with the formula: Length × Width^2 × 0.5236.

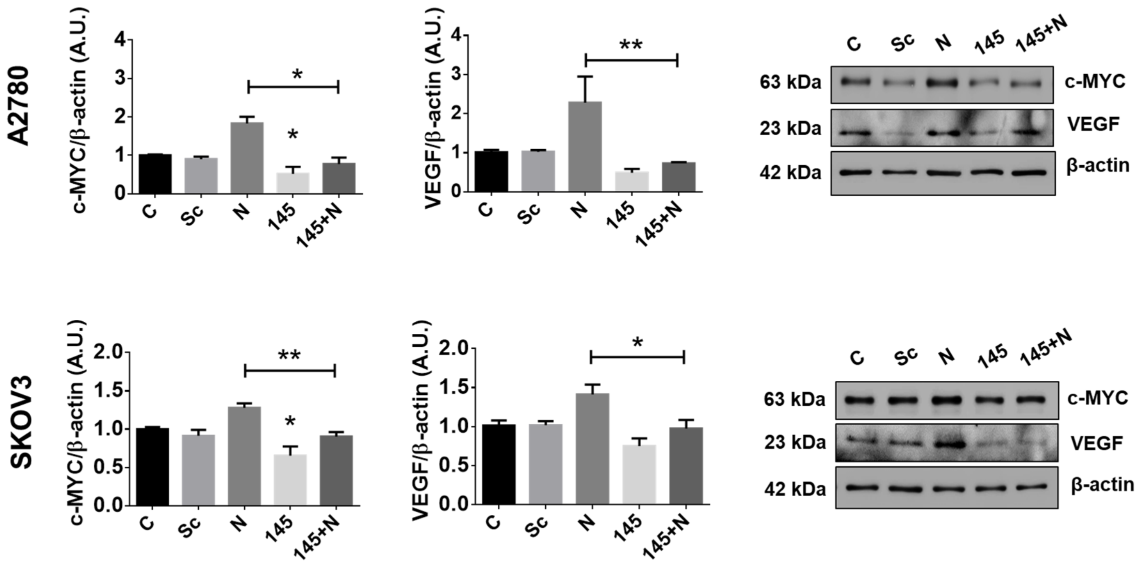

Figure S4. Detection of c-MYC and VEGF levels in EOC cells by western-blot. A2780 and SKOV3 cells were transfected with miR-145 and stimulated with NGF (3 h, 100 ng/mL for A2780 cells and 150 ng/mL for SKOV3 cells). Protein extracts (50 ug) were used to perform the electrophoresis. The assay was performed using the following antibodies: mouse monoclonal anti-VEGF (Abcam ab1316, 1:200 in TTBS, band shown corresponds to VEGF165) and rabbit monoclonal c-MYC antibody (Abcam ab32072, 1:500 in milk/TTBS 5%). The incubation was overnight at 4 °C. N = 4 independent experiments. C: control condition (cells treated only with lipofectamine), SC: cells transfected with the scrambled sequence, N: cells treated with lipofectamine and stimulated with NGF. 145: cells transfected with miR-145. * = $p < 0.05$ and ** = $p < 0.01$, compared with each indicated condition (Kruskal Wallis test and Dunn's post-test).
